# Antioxidant Systems, lncRNAs, and Tunneling Nanotubes in Cell Death Rescue from Cigarette Smoke Exposure

**DOI:** 10.3390/cells11152277

**Published:** 2022-07-23

**Authors:** Jose Lorenzo M. Ferrer, Reynaldo L. Garcia

**Affiliations:** Disease Molecular Biology and Epigenetics Laboratory, National Institute of Molecular Biology and Biotechnology, University of the Philippines Diliman, Quezon City 1101, Philippines; jmferrer2@up.edu.ph

**Keywords:** redox, oxidative stress, ROS, antioxidant systems, long non-coding RNAs, tunneling nanotubes

## Abstract

Cigarette smoke is a rich source of carcinogens and reactive oxygen species (ROS) that can damage macromolecules including DNA. Repair systems can restore DNA integrity. Depending on the duration or intensity of stress signals, cells may utilize various survival and adaptive mechanisms. ROS levels are kept in check through redundant detoxification processes controlled largely by antioxidant systems. This review covers and expands on the mechanisms available to cigarette smoke-exposed cancer cells for restoring the redox balance. These include multiple layers of transcriptional control, each of which is posited to be activated upon reaching a particular stress threshold, among them the *NRF2* pathway, the *AP-1* and *NF-kB* pathways, and, finally, *TP53*, which triggers apoptosis if extreme toxicity is reached. The review also discusses long noncoding RNAs, which have been implicated recently in regulating oxidative stress—with roles in ROS detoxification, the inflammatory response, oxidative stress-induced apoptosis, and mitochondrial oxidative phosphorylation. Lastly, the emerging roles of tunneling nanotubes in providing additional mechanisms for metabolic rescue and the regulation of redox imbalance are considered, further highlighting the expanded redox reset arsenal available to cells.

## 1. Introduction

Reactive oxygen species (ROS) are free radicals generated by cells as a consequence of normal metabolism. They play a role in intracellular signaling but have the potential to cause damage to cells and macromolecules if produced and made available in excess [1,2]. ROS levels are kept in check through redundant detoxification processes controlled largely by antioxidant systems. There are several potential endogenous sources of ROS including—but not limited to—mitochondrial oxidative phosphorylation, cytochrome p450 metabolism, peroxisomes, and activation of inflammatory cells such as macrophages and neutrophils [3]. Exogenous sources include cigarette smoke, inhaled pollutants, ultraviolet radiation, microbial infections, and even allergens, among others [4,5,6,7,8,9].

Many free radicals and oxidants exist in a steady state in the gas phase of cigarette smoke, while superoxide anion (O_2_^•−^), H_2_O_2_, and the reactive hydroxyl radical (HO^•^) can be produced from some of the water-soluble components of the cigarette. These substances are known to cause oxidative damage to cellular lipids, proteins, and, in particular, DNA [10,11]. Well-known mediators of DNA damage, these ROS are able to induce double-strand breaks (DSBs) and the oxidation of nucleoside bases (e.g., 8-oxo guanine formation), the latter of which can cause C-A or G-T substitutions as well as the generation of DSBs if base excision repair fails [12,13,14]. Moreover, these oxidized bases also physically hinder replication forks [15], causing DSBs and under-replicated or over-replicated DNA, increasing the likelihood of mutagenesis as well as genomic instability [16]. 

ROS play an enabling role in tumorigenesis. Their production is elevated in cancer cells as a consequence of mutations in oncogenes and tumor suppressors, and the resulting dysregulated signaling pathways, increased metabolic rate, and hypoxic conditions. Cancer cells then have to adjust their redox balance with the help of antioxidant systems in order to support the pro-proliferative program while avoiding cell death. Cancer cells, however, have to endure oxidative stress throughout their journey from initiation to metastatic spread—upon detachment from the extracellular matrix (ECM); as they intravasate, pass through circulation, and extravasate to new sites; or even during disease recurrence [2,17,18].

As cancer progresses, the interaction between tumor cells and their microenvironment further adds to the oxidative stress. Cooperation among tumor cells, cancer-associated fibroblasts (CAFs), and tumor-associated macrophages (TAMs) has been shown to result in ROS-stimulated migration and anchorage-independent growth, as well as immunosuppression [17,18]. Cancer cells therefore have to invoke adaptive mechanisms to ensure ROS levels are kept within an acceptable range [2]. 

During metastasis, cancer cells employ other strategies to ensure their survival, including movement through the blood in clusters, either among themselves or with neutrophils. Clustering has been shown to aid in their ability to successfully metastasize compared to single circulating cancer cells, and minimizes their exposure to oxygen, thereby reducing the production of mitochondrial ROS. [19,20,21,22]. E-cadherin has also been shown to aid in the survival of metastasizing breast cancer cells by limiting oxidative stress, although the mechanism is not entirely known. While E-cadherin is known to promote cell–cell interaction, its deletion did not affect the movement of cancer cells in clusters [23]. Whether the above evasion mechanisms are universally employed by other cancer cell types merits further investigation. 

This review article covers and expands on the mechanisms available to cigarette smoke-exposed cancer cells for restoring the redox balance that helps prevent them from triggering apoptosis. In particular, the role of long noncoding RNAs (lncRNAs) in helping regulate ROS levels and in promoting or averting the oncogenic program is discussed. These non-coding RNAs >200 bp in length are pervasively transcribed in most cells and tissues and have been shown to play roles in transcriptional regulation, translational regulation, and histone and DNA methylation, and as miRNA sponges or precursors to miRNAs and siRNAs [24]. This review also explores the emerging roles of tunneling nanotubes (TNTs)—filamentous actin-based structures implicated in direct cell-cell communication—and TNT-based supercellularity in the following: maintaining homeostasis amidst oxidative stress; sustaining oncogenic phenotypes and metastatic spread through long-distance intercellular transfer of cargoes; the development of chemoresistance; and the rescue of cells from toxicity and cell death through the redistribution of stress factors [25]. To provide a more comprehensive coverage of the research topic, the role of antioxidant systems and graded cell adaptive responses are also discussed. This review does not intend to cover the field in its entirety but aims to add to the growing list of ROS rescue mechanisms available to cells by way of an analysis of representative studies. 

## 2. Cigarette Smoke and Oxidative Stress

### 2.1. Genetic and Epigenetic Effects

Cigarette smoke is a rich source of known and suspected carcinogens, ROS, and reactive nitrogen species (RNS) that can damage macromolecules including nucleic acids, proteins, and lipids. Its carcinogenic components include, among others, polycyclic aromatic hydrocarbons, aromatic amines, tobacco-specific nitrosamines, and phenolic compounds [26]. The gas phase of cigarette smoke produces ROS during combustion of the tobacco and is inhaled during smoking [27,28]. Particulates in cigarette smoke can also accumulate in the lungs as a layer of tar, and, in aqueous solution, can produce oxidative agents via redox cycling reactions. The role of smoking-induced oxidative stress in inflammation is now widely acknowledged, with inflammatory reactions themselves further generating ROS. Although some other components of cigarette smoke, such as metals, also contribute to oxidative stress [29,30], many of its carcinogenic components do not, but are still able to cause damage via different mechanisms.

Chronic smoking is an established risk factor for the development of lung cancer and other malignancies, which can be attributed to the damage it causes to both the genome and epigenome. Oxidative damage secondary to cigarette smoke exposure can lead to the direct oxidation of a base in the DNA, and/or to the misincorporation of the oxidized deoxynucleoside triphosphates into the growing chain by the DNA polymerases [31,32,33]. If DNA repair is faulty, erroneous replication ensues and the mutation is propagated [31,34]. If the DNA damage happens in regions of the genome harboring protooncogenes or tumor suppressors, their activation or inactivation, respectively, may signal oncogenesis [35]. The role of functioning DNA repair systems in restoring DNA integrity—after exposure to toxic insults such as carcinogens, ROS, and RNS from cigarette smoke—is therefore paramount. More recently, DNA damage secondary to chronic cigarette smoke exposure has been shown to activate PARP-1, the key mediator of the parthanatos pathway—a programmed cell death pathway characterized by regulated necrosis with a consequent rupture of cells and organelles leading to inflammation [36].

Components of cigarette smoke may also exert damaging effects on the epigenome via different mechanisms: (1) Its toxic components may directly damage DNA and may thus affect the binding of DNA methyltransferases (DNMTs) [37]; (2) Faulty DNA repair pathways can have similar effects and lead to hypomethylation [35,38]; (3) The deoxyguanosine derivative 8-oxo-2’-deoxyguanosine (8-oxo-dG), a product of oxidative damage—if formed at guanine residues in CpG dinucleotides—can inhibit the methylation of cytosine [39]; (4) Unrepaired 8-oxodG can also lead to erroneous replication by inducing G to T transversions and thus contribute to net CpG dinucleotide loss [40]; Lastly, other components of cigarette smoke act as direct inhibitors of DNMTs [41,42,43]. Thus, the exposure of various cells to cigarette smoke or its components is often associated with a global decrease in 5-methylcytosine and a corresponding increase in 5-hydroxymethyl cytosine levels [44,45,46].

### 2.2. Adaptive Responses to Acute and Chronic Oxidative Stress

While cigarette smoke exposure can instigate a program of carcinogenesis via different mechanisms, the consequences may not be immediately disastrous to cells, which can, in fact, adapt to the toxic insults caused by carcinogens and oxidative stress. The journey of cancer cells from a primary site to a new metastatic niche is hostile and constantly accompanied by redox imbalance and adjustments. Cells have to invoke short-term adaptive responses and the activation of genetic programs in response to acute and chronic oxidative stress, respectively [2]. By controlling ROS levels or developing a resistant phenotype, cancer cells ensure their survival, and are able to proliferate and metastasize.

Metabolic rerouting is a short-term adaptive response to oxidative stress. For instance, at non-toxic threshold levels of H_2_O_2_, cells activate glucose-6-phosphate dehydrogenase (G6PD) and reroute glucose metabolism from glycolysis through the oxidative arm of the pentose phosphate pathway (PPP) toward nucleotide synthesis. This allows for the increased reduction of nicotinamide adenine dinucleotide phosphate (NADP+) to NADPH (reduced NADP) [47], an important metabolite in the reductive biosynthesis of macromolecules—making it an indispensable component of the cell’s antioxidant arsenal [2]. Increased NADPH then allows glutathione reductase 1 (GSR1) and thioredoxin reductase 1/2 (TXNRD1/2) to bring down ROS to homeostatic levels via the augmentation of glutathione- and thioredoxin-based antioxidant systems [2]. Acute exposure to oxidative stress may also activate the PI3K/Akt pathway by inhibiting its negative regulator PTEN through Cys-124. As a consequence, antioxidant genes are upregulated and cells survive [48,49].

Chronic oxidative stress requires cells to develop a more resistant phenotype via the activation of genetic programs. In this regard, incessant exposure to cigarette smoke provides a good model system for demonstrating the functional sequelae of redox imbalance. Cigarette smoke contains thousands of different components, including nicotine, N-nitrosonornicotine (NNN), and nicotine-derived nitrosamine ketone (NNK), that can damage cellular components and upregulate various transcription factors, oncogenes, and receptors [50,51].

#### 2.2.1. Graded Adaptive Responses: Multi-Level Transcriptional Control

Different layers of transcriptional control are accessed by cells to reset their redox status, depending on how overwhelmed the antioxidant mechanisms have become. A widely held hypothesis posits that cells respond to oxidative stress in a graded fashion. The transcription factor NRF2 (nuclear factor erythroid 2-related factor 2), which regulates a panoply of antioxidant and detoxification genes, is considered the first-line inducible defense against modest increases in ROS/RNS [52,53]. AP-1 and NF-κB, among other transcription factors that are activated by higher ROS/RNS levels, are considered to be a second-line defense. Lastly, activated in response to excessive ROS levels, TP53 constitutes the final line of defense and can induce apoptosis if toxic levels are achieved [54]. 

As main regulator of intracellular redox homeostasis, NRF2 (with the help of the musculoaponeurotic fibrosarcoma or MAF proteins) transactivates a long list of antioxidant genes upon exposure to ROS or soft electrophiles [52,55] by binding to the antioxidant response elements (ARE) of target genes such as heme oxidase 1 (HMOX1), NAD(P)H quinone dehydrogenase 1 (NQO1), and glutathione-S-transferases (GST), as well as long non-coding RNAs (lncRNAs), such as the smoke- and cancer-associated lncRNA 1 (SCAL1) [56,57]. In the above model, an ROS threshold, whereby the antioxidant systems are overwhelmed, must be breached before other antioxidant transcription factors are activated. NRF2 may induce and invoke the help of Krupple-like factor 9 (KLF9) to downregulate the antioxidants thioredoxin reductase 2 (TXNRD2) and peroxiredoxin 6 (PRDX6) [58,59]. Under normal conditions, KEAP1 (Kelch ECH associating protein 1) represses NRF2 by binding to it and promoting its proteasomal degradation [60]. 

Components of cigarette smoke, as well as endogenous chemicals generated upon cigarette smoke exposure, activate the NRF2–KEAP1 pathway by modifying their critical cysteine residues, or by inducing endoplasmic reticulum (ER) stress, thereby activating the unfolded protein response (UPR) [61]. Chemical inducers of ARE genes can cause structural alterations of the NRF2/KEAP1/CUL3 complex by modifying the cysteine thiols of NRF2 and KEAP1, a consequence of which is the inhibition of NRF2 ubiquitination. The role of NRF2 in cancer is context dependent. On the one hand, it is able to mediate the protective effects of chemopreventive drugs [62]; on the other, it can also promote tumor growth and resistance to oxidants and anticancer agents [63].

The NRF2 transcript is expressed ubiquitously although at varying levels depending on the cell type. Further, it is expressed independently of inducers, and may suggest post-transcriptional regulation to promote its activation. Several studies suggest the involvement of microRNAs in the posttranscriptional regulation not only of NRF2, but also of the KEAP1 and MAF genes. Mature microRNAs (miRNAs) are short (18–24 base pairs), genomically encoded noncoding RNAs that normally act on 3′ untranslated regions (3′ UTR) of target mRNAs causing either transcript degradation or translational repression [64,65]. In silico analyses by Papp et al. [66] indicate that dozens of microRNAs are predicted to bind the NRF2 3′UTR, with 63 of them serving as potential feedback loops. A consequence of NRF2 downregulation by miRNAs is altered ARE-mediated redox signaling [67]. KEAP1 is itself a target of miRNA regulation. MiR-200a has been shown to target KEAP1 mRNA in the human breast cancer cell lines MDA-MB-231 and Hs578T. This leads to enhanced activation of NRF2 and upregulated ARE-mediated antioxidant gene expression. The knockdown of the miR-200a function, on the other hand, correlated with KEAP1 derepression and diminished NRF2 levels [68]. Because of their role in finetuning redox regulation, this subset of miRNAs have been referred to as redoximiRs [69].

The NFKB family of transcription factors is regulated by oxidant-sensitive molecular targets [70,71]. NF-κb is said to be a complex hub and master regulator of many crucial signaling cascades, especially those involved in inflammation and immunity but also in cell growth, differentiation, development, and apoptosis [72]. In most cases, NFKB promotes the expression of genes involved in cellular survival [73]. In the context of ROS, the NFKB pathway is expected to counteract ROS by targeting apoptosis-associated signaling cascades in order to keep the cell alive [74]. One of the main ROS- and apoptosis-related signaling pathways that intersects with NFKB is the JNK pathway, which promotes programmed cell death. NFKB inhibition results in an accumulation of ROS and sustained activation of the JNK pathway leading to apoptosis or necrosis [75,76,77].

The JNK cascade also interacts with the Activating Protein-1 (AP-1) transcription factor, which is a heterodimeric leucine zipper complex consisting of the Fos and Jun proto-oncogene proteins [78]. Upon AP-1 induction by pro-inflammatory cytokines and genotoxic stress, AP-1 proteins bind to the TPA (12-O-tetradecanoylphorbol 13-acetate)-response element (TRE) to facilitate transcriptional activation of many genes [79], particularly those that belong to the JNK and p38 MAPK pathways [80]. The redox regulation of Fos and Jun is mediated through their DNA binding domains. Specifically, they contain a conserved cysteine residue that can be oxidized/chemically modified, resulting in the inhibition of AP-1 DNA binding. It has an opposite effect on AP-1 when this cysteine residue is reduced either chemically or by Ref-1, a nuclear redox factor [81]. Zhong et al. showed that tobacco smoke activates AP-1 through all four distinct MAPK pathways including ERK1/2, JNK, p38, and ERK5. The authors then showed that smoking upregulates the expression of AP-1 dependent cell cycle (i.e., Cyclin D1, PCNA) and cell differentiation proteins (i.e., Keratin 5, Keratin 14), highlighting the importance of the MAPK/AP-1 signal pathway in smoke-induced tumorigenesis [82]. Notably, AP-1 is also activated by an increase in H_2_O_2_ levels, which also affect the expression of hypoxia-inducible factor 1 alpha (HIF1A), thought to be a key factor in hypoxia [83,84]. 

Hypoxia is a key consideration in the pathology of cancer and other human diseases, since low oxygen levels create a unique microenvironment that affects the activity of many signaling pathways. This state of low oxygen levels affects the activity of the cytochrome chain responsible for mitochondrial oxidative phosphorylation, simultaneously resulting in a decrease in ATP synthesis and increased ROS as well as a decrease in the activity of the cellular antioxidant system, thus potentially leading to oxidative stress [85,86,87,88,89]. Despite being clearly upregulated in hypoxic conditions, HIF1A was also shown to be upregulated under normoxia in response to various growth factors that also stimulate the generation of ROS [90,91,92,93,94]. In their work, Daijo et al. showed that, in human lung epithelial-like cells under non-hypoxic conditions, a cigarette smoke extract induced a time- and concentration-dependent accumulation of HIF1A protein, consequently upregulating two factors: vascular endothelial growth factor (VEGF) and regulated in development and DNA damage response 1 (REDD1) [95]. The latter is a stress response protein, which, like VEGF, is involved in smoking-induced emphysematous changes [96].

TP53 can switch gears depending on the level of oxidative stress. Under mild conditions, TP53 contributes to cellular adaptation by inducing antioxidant gene expression. It transactivates genes coding for ROS scavengers, supports glutathione synthesis, increases the production of NADPH, and downregulates the expression of nitric oxide synthase (NOS2) and cyclooxygenase 2 (COX2)—two key pro-oxidant enzymes [97,98]. Under more stringent conditions, TP53 is able to orchestrate apoptosis by stimulating the production of ROS. Under pro-apoptotic conditions, TP53 is also able to downregulate SOD2 (superoxide dismutase 2) [99]—a key antioxidant enzyme—and NRF2-targeted genes [100]. ROS employ redundant mechanisms to induce apoptosis. They can activate the intrinsic mitochondrial pathway, the extrinsic death receptor pathway, and the endoplasmic reticulum (ER) stress pathway [101]. Further, ROS can trigger ferroptosis, an iron-dependent form of cell death [102].

#### 2.2.2. Oxidative Stress-Upregulated Oncogenes 

An important cigarette smoke extract (CSE)-induced oncogene is β-catenin, a key regulator of cell cycle, adhesion, development, and tumor formation [103,104,105]. β-catenin is an integral component of the Wnt signaling pathway and regulates the expression of a multitude of genes by entering the nucleus and binding T cell factor/lymphoid enhancer factor (TCF/LEF) transcription factors [106]. It has also been shown to interact with the forkhead box, class O (FOXO) family of transcriptional regulators at low levels of oxidative stress [107]. FOXO family members are important regulators of the cellular stress response and promote cellular antioxidant defense [108]. FOXO is able to compete with TCF for interaction with β-catenin, which leads to enhanced FOXO transcriptional activity. This equates to a protective response that inhibits cell cycle progression, thereby allowing cells to manage oxidative damage effectively [107]. Increased levels of ROS, however, lead to a different readout. They promote cellular proliferation and transformation [109] that are partly mediated via the nucleoredoxin (NRX)-dependent inhibition of WNT/β-catenin signaling [110]. This classifies β-catenin as a key regulator that senses whether cells should proliferate or arrest to repair oxidative damage [111]. The ROS H_2_O_2_ can promote β-catenin stabilization [110]. However, the role of ROS in promoting or inhibiting WNT signaling is generally believed to be cell- and tissue-specific [112].

Another CSE-regulated oncogene is MYC [113], a transcription factor often found to be overexpressed in human malignancies resulting in uncontrolled proliferation [114,115,116,117]. In the study by Lu et al., CSE increased the levels of MYC in human bronchial epithelial cells, resulting in a greater invasion and migration of transformed cells [113]. Through ChIP assays, they further demonstrated that, as a consequence of the upregulation of MYC, CCAT1 expression also went up, since MYC binds to the CCAT1 promoter [113]. CCAT1 is an lncRNA first found to be upregulated in colon cancer but now known to be involved in other cancers as well [118,119,120,121,122]. Lastly, let-7c—a tumor suppressor miRNA— was shown to negatively regulate c-Myc, while CCAT1 is able to derepress c-Myc expression by sponging let-7c, thus creating a positive feedback loop to promote CSE-induced CCAT1 and MYC expression [113].

Oxidative stress-induced growth inhibitor 1 (OSGIN1) is also a CSE-associated factor that is markedly upregulated in the airway epithelia of smokers compared to nonsmokers [123]. The short OSGIN1-52 kDa isoform is regulated by p53 and is induced by DNA damage. It regulates apoptosis by inducing cytochrome c release from mitochondria [124]. The OSGIN1-61 kDa isoform, on the other hand, was shown to be an NRF2 transcriptional target in human astrocytes [125]. This is consistent with its reported role in reducing oxidative stress [126]. Whether it has a similar role in lung tissues remains to be established.

#### 2.2.3. Upregulated Receptors in Oxidative Stress Response

Several receptors are also key components in the response of cells to changes in the cellular redox status, such as in cigarette smoking. Nicotinic acetylcholine receptors (nAChRs) are hetero- or homopentamers that can be classified into neuronal or muscle nAChRs, with each having a different combination of subunits. As their name suggests, nAChRs are bound and regulated by nicotine, as well as its derivatives NNK and NNN, both of which may bind to the receptors with higher affinity [127]. These induced nAChRs, specifically the homomeric NACHRA7, can activate several downstream transcriptional programs involved in cell proliferation, metastasis, angiogenesis, and resistance to apoptosis in cancer [128,129,130,131].

The functional significance of nAChR upregulation includes the idea that nicotine exposure leads to both higher nicotine sensitivity and greater nAChR function, which may be explained by increased cell surface trafficking of nAChRs and enhanced receptor assembly and/or maturation [132]. Smokers generally have greater expression levels of NACHRA7 than non-smokers, and different nAChR subunits are found to be expressed in the NSCLC cells of smokers and non-smokers [133]. An elevated expression of NACHRA7 was observed to be induced by nicotine in squamous cell carcinoma of the lung (SCC-L) cell lines [134]. These findings provide evidence for the role of NACHRA7 upregulation in nicotine-induced tumorigenesis.

Previous studies have shown that polymorphisms in the chromosome region containing three nAChR genes (CHRNA3, CHRNA4, and CHRNA5) are associated with an increased cancer risk [135]. Additionally, CHRNA7 gene duplication is associated with poor prognoses in lung cancer and chronic obstructive pulmonary disease (COPD) [136]. In fact, by binding to these increased numbers of receptors, nicotine can activate pathways that could contribute to carcinogenesis, possibly by the NACHRA7-mediated modulation of the inflammatory response [137,138]. Moreover, in melanoma, nicotine has been demonstrated to mediate Programmed Cell Death Ligand 1 (PD-L1) expression via CHRNA9, promoting cell migration and proliferation [139]. PD-L1 suppression has been shown to promote an effective immune response against cancer cells [140]. A recent study has identified NACHRA7 as the receptor through which CSE could increase PD-L1 levels. In particular, to regulate PD-L1, NACHRA7 was shown to regulate both the STAT3 and NRF2 pathways, showing that NACHRA7 may also have a role in the cellular response to oxidative stress. Nicotine has been shown to increase ROS levels [141]. By inducing ROS generation and activating NFKB via nAChRs, nicotine was shown to cause apoptosis in renal proximal tubular epithelial cells [142,143].

Interestingly, in PC12 cells, ethanol-induced intracellular oxidative stress was reduced after selectively activating NACHRA7 with 3-(2,4)-dimethoxybenzylidine anabaseine, a receptor agonist. This suggests that NACHRA7 may prevent ROS accumulation [144]. Additionally, in the same cell line, another study demonstrated that nAChR activation via nicotine, albeit only at low concentrations, inhibited lipid peroxidation and rescued lower rates of cell viability upon H_2_O_2_ and Abeta treatment [145]. Several studies performed in a neuronal context also support the potential antioxidant and cytoprotective role of NACHRA7. In an in vitro mouse model of neuroinflammation, treatment with GTS21, a partial NACHRA7 agonist, significantly reduced the LPS-mediated secretion of inflammatory cytokines, inhibited LPS-mediated NFKB nuclear translocation, and upregulated canonical NRF2 antioxidant genes [146]. On the other hand, in SH-SY5Y cells, the siRNA-mediated knockdown of NACHRA7 increased lipid peroxidation and stimulated toxicity induced by Abeta, which is key factor in Alzheimer’s disease pathogenesis [147].

## 3. ROS Scavengers: Enzymatic, Non-Enzymatic and Indirect Antioxidant Systems

The previous section described examples of the dysregulation of integral signaling pathways and/or genes due to the oxidative stress induced by cigarette smoke or CSE. To combat the harmful effects of excess ROS, cellular antioxidant systems exist in aerobic organisms and humans [148]. These systems are divided into two categories: enzymatic and non-enzymatic. Enzymatic systems are able to repair damaged DNA and proteins, fight oxidized lipids, stop the chain propagation of peroxyl lipid radicals, and repair damaged cell membranes and molecules [149]. On the other hand, non-enzymatic antioxidants mainly involve small molecules that are able to rapidly inactivate radicals and oxidants [150]. A few other antioxidants exert their function indirectly.

### 3.1. Enzymatic Antioxidant Systems

Superoxide dismutases (SODs) comprise one of the most essential enzymatic antioxidant systems in the lungs. Superoxide anions are considered the primary cellular ROS, given that they initiate a chain of reactions to create “secondary” ROS [151,152]. SODs, discovered over 50 years ago, are thought to be the first line of defense against oxygen free radicals and have three forms that are widely expressed in the human lung: cytosolic copper/zinc superoxide dismutase (CuZn-SOD, or SOD1), mitochondrial manganese Mn-SOD (SOD2), and extracellular EC-SOD (SOD3) [153,154,155]. The first two SOD classes serve as bulk scavengers of superoxide radicals while EC-SOD is believed to protect the lung matrix [156]. The function of SODs is to convert superoxide into hydrogen peroxide [157], which easily diffuses across the plasma membrane and can be processed by another major enzymatic antioxidant: catalase [153]. Both SOD1 and SOD2 exhibit a tumor suppressor activity but they may also be upregulated during tumorigenesis [2,158].

The first antioxidant enzyme to ever be studied, catalase (CAT), is a ubiquitous heme-containing tetramer that catalyzes the dismutation of two H_2_O_2_ molecules produced by SODs or other oxidases into oxygen and water [159]. The degradation of H_2_O_2_ is accomplished via the conversion between 2 conformations of catalase-ferricatalase (iron coordinated to water) and compound I (iron complexed with an oxygen atom). Under high energy demand there is a quick accumulation of H_2_O_2_, so this degradation occurs in an energy-efficient manner. In general, CAT activity can be intensified or diminished depending on various parameters of environmental stress, including its duration, intensity, and type. It is believed that stimuli that lower protein turnover also reduce CAT activity.

Other enzymes that scavenge H_2_O_2_ include periredoxins (PRDXs) and glutathione peroxidases (GPXs), which reduce H_2_O_2_ to H_2_O as well as lipid hydroperoxides resulting from membrane lipid peroxidation [160,161]. As well as regulating ROS levels, PRDXs and GPXs can also counter the activity of RNS by helping eliminate nitric oxide, as well as contributing to the reduction of peroxynitrite anion, and protein denitrosylation [162]. Reduced glutathione (GSH) itself is considered a non-enzymatic antioxidant. GSH donates its electron to H_2_O_2_ to reduce it into H_2_O and O_2_. Oxidized glutathione (GSSG) is reduced into GSH by GSH reductase, which uses NAD(P)H as the electron donor [163]. GPXs are also important for the protection of cell membranes from lipid peroxidation [164].

### 3.2. Non-Enzymatic Antioxidants

Non-enzymatic antioxidants rely on lower molecular mass substances and strategic localization for defense against ROS [150]. Aside from the aforementioned GSH, these include metal-binding proteins (MBPs), vitamin E, vitamin C, uric acid, and selenium [150,165]. 

Albumin (ALB), ceruloplasmin, myoglobin (MB), ferritin, and transferrin are some key members of the first example of non-enzymatic antioxidants, the metal-binding proteins (MBPs) [166,167,168,169]. Being the main contributors to the plasma antioxidant capacity, MBPs naturally bind potentially pro-oxidant transition metal ions such as Cu^2+^ and Fe^2+^, which can react with H_2_O_2_ to produce more ROS via the Fenton reaction. Some of these proteins can additionally act as true scavengers of reactive species: e.g., free sulfhydryl groups of cysteine in ALB and metallothioneins (MTs) are able to scavenge hydroxyl radicals. Such MBPs as transferrin (TF), ferritin (FER), and lactoferrin (LTF) are chelators of redox-active iron (Fe^2+^), which can be effective free radical inhibitors in the Fenton reaction [166,167,168,169]. In contrast, ceruloplasmin acts as a reactive species inhibitor by binding free copper (Cu^2+^) and iron ions (Fe^2+^), or as a chain-breaking antioxidant [170,171,172]. In turn, ALB is a multifunctional antioxidative protein, which binds redox metals (Fe II and Cu II) and can also act as a true scavenger by reacting with hydroxyl radicals [173,174,175,176]. Myoglobin is another MBP, which is mainly an effective NO scavenger [177]. 

A pair of exogenous vitamins, Vitamin C and Vitamin E, also contribute to free radical regulation. Vitamin E, also referred to as α-tocopherol, which is its prevalent species, oxidizes lipid peroxyl radicals, which are made during lipid peroxidation [178]. Being lipid-soluble, Vitamin E is found in the hydrophobic region of the plasma membrane and functions to protect the lipid bilayer from oxidants [179]. Moreover, vitamin E also promotes cancer cell programmed cell death [180]. In comparison, Vitamin C, commonly known as ascorbic acid, is water-soluble and scavenges oxygen free radicals intra- and extracellularly [181]. In fact, it is able to restore Vitamin E free radicals to vitamin E [182].

In addition, uric acid (UA) is a key aqueous antioxidant in humans [183,184]. Generated through purine metabolism, UA selectively scavenges peroxynitrite in conjunction with Vitamin C and thiols but notably cannot scavenge superoxide [185]. 

Finally, selenium, an essential trace element, was found to be a key component of several antioxidant enzymes such as GPx, TXNRD, and iodothyronine deiodinases [186]. Humans are estimated to have about 25 Se-containing proteins (selenoproteins) [187].

Other non-enzymatic antioxidants include melatonin, bilirubin, and polyamines [188,189,190]. 

### 3.3. Indirect Antioxidants

Per se, sestrins (SESN1, 2, and 3) lack an intrinsic catalytic antioxidant activity. Their antioxidant action, however, is executed in one of two ways: (1) They promote the autophagic degradation of KEAP1 and thus upregulate NRF2 signaling and antioxidant gene expression; (2) They block mTORC1 activation, effectively attenuating reactive oxygen species accumulation [191].

Drug-metabolizing enzymes can also be considered as indirect-acting antioxidants. Glutathione transferases [192], aldo-ketoreductases [193], carbonyl reductases [194], aldehyde dehydrogenases [195], and UDP-glucuronosyltransferases [196] belong to this group. They help prevent quinones and hydroquinones from redox cycling, and help prevent electrophiles and lipid peroxidation products from depleting reduced glutathione [2].

Sirtuin 3 (SIRT3) is also an indirect antioxidant. It can increase the scavenging of superoxides in mitochondria by catalyzing the deacetylation of SOD2. Further, it can increase the generation of NADPH in mitochondria by catalyzing the deacetylation of IDH2 [197]. NADPH plays an important role in antioxidant defenses and is a key metabolite in the reductive biosynthesis of macromolecules [2].

**Figure 1:**
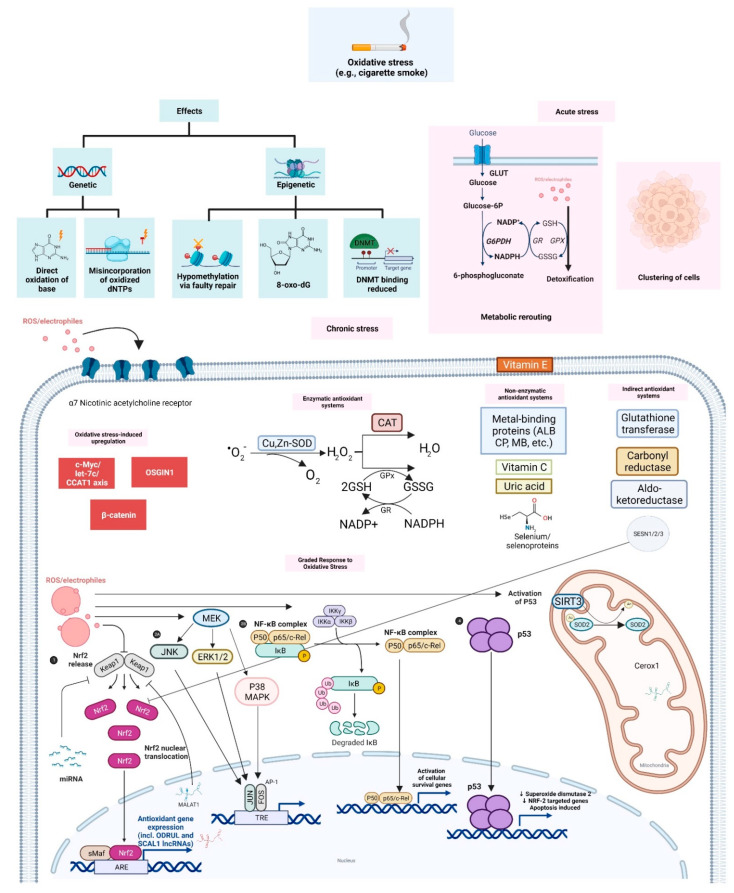
Schematic view of the cellular effects and responses of cells to sources of oxidative stress (including cigarette smoke exposure). Cigarette smoke exposure causes several genetic and epigenetic alterations. Depending on the length and/or intensity of stress exposure, cells utilize various survival and adaptive mechanisms. Under acute stress, cells undergo metabolic rerouting, resulting in increased NADPH, which is useful for downstream ROS detoxification. Cancer cells employ clustering, a mechanism to minimize oxygen exposure and thus the production of mitochondrial ROS. On the other hand, chronic oxidative stress requires the activation of various antioxidant systems and genetic programs. Furthermore, non-coding RNAs such as miRNAs (e.g., miR-200A) and lncRNAs (e.g., CEROX1, SCAL1, ODRUL, and MALAT1) are increasingly thought to be involved in the cellular redox response and/or in ROS detoxification.

The major survival and adaptive responses invoked by cells in response to varying levels of oxidative stress are summarized in Figure 1 below.

## 4. Long Non-Coding RNAs and Oxidative Stress

MiRNAs are not the only species of non-coding RNAs that are involved in the cellular response to environmental stressors. Similar to redoximiRs, various lncRNAs have been associated with the dysregulation of the NRF2–KEAP1 pathway. LncRNAs are non-coding RNAs >200 base pairs in length and are pervasively transcribed from the human genome, with roles in chromatin remodeling as well as the transcriptional and post-transcriptional regulation of genes [198,199]. Our recent work on the lncRNA SCAL1, which is directly regulated by NRF2 and was discovered by Thai et al. [56], suggests a protective role for SCAL1 in the context of cigarette smoke exposure—with the caveat that cells with damaged DNA are also allowed to survive and multiply [57]. Our data show that SCAL1 mediates ROS detoxification in A549 cells, aside from promoting cell migration, extensive cytoskeletal remodeling, and resistance to apoptosis [57].

Another redox regulator lncRNA is the hydrogen peroxide-induced Metastasis Associated Lung Adenocarcinoma Transcript 1 (MALAT1). MALAT1 carries out its protective function by downregulating KEAP1, resulting in the stabilization of NRF2 [200]. It also downregulates the expression of several miRNAs by acting as a sponge, thereby influencing the inflammatory response and consequently the redox status of cells, which, when perturbed, also affects MALAT1 expression especially in hypoxia or ischemia [201,202].

Several other lncRNAs interact with NRF2, albeit with dissimilar readouts [79,203,204,205]. One such ncRNA is osteosarcoma doxorubicin resistance-related up-regulated lncRNA (ODRUL) [206]. In their experiments, Gao et al. used silver nanoparticles (AgNP) to incur cytotoxicity to erythroid cells by generating ROS/oxidative stress coming from Ag particles or ions. The authors discovered that ODRUL promoted this AgNP-induced toxicity [206]. Specifically, under stress caused by the metal particles, NRF2 promotes the expression of ODRUL, which then inhibits the pro-survival pathway PI4KA-AKT-BCL2 and enhances JNK inhibition of BCL2 via the PI4KA-AKT-JNK cascade [206].

Similarly, Li et al. showed that, in both in vitro and in vivo models of hypoxic pulmonary hypertension (HPH), the lncRNA myocardial infarction-associated transcript (MIAT) is upregulated when compared with normoxic conditions [207]. MIAT was shown to contribute to the proliferative and migratory abilities of human pulmonary artery endothelial cells (HPAECs) via its targeted downregulation of miR-29a-5p and thus the inhibition of the NRF2 pathway [207]. A similar story is seen with the lncRNA forkhead box D3 antisense RNA 1 (FOXD3-AS1), which was discovered in hyperoxia-induced lung injury in mice and was demonstrated to mediate apoptosis induced by oxidative stress [208]. In human lung epithelial cells, this lncRNA was shown to sequester the cytoprotective miR-150, contributing to programmed cell death in the context of hyperoxic stress.

Wang et al. also found that H19 and HULC are differentially expressed lncRNAs in bile duct cancer cell lines that were subject to treatment with H_2_O_2_, glucose oxidase, and other hypoxic or inflammatory factors [209]. Through the inflammation pathway, these lncRNAs were shown to promote migration and invasion. Mechanistically, H19 and HULC both acted as competitive endogenous RNAs (ceRNAs) by sponging let-7a/let-7b and miR-372/miR-373, respectively. These miRNAs are known to regulate crucial inflammatory factors, particularly the chemokine receptor CXCR4 and the cytokine IL-6 [209]. The authors suggest the presence of a positive lncRNA-activated feedback loop between inflammation and oxidative stress that might promote oncogenesis.

Remarkably, the unusually abundant mouse cytoplasmic lncRNA Cerox1 was the first lncRNA demonstrated to regulate mitochondrial oxidative phosphorylation, which, when coupled with the mitochondrial electron transport chain, is able to generate the majority of the ATP required by the cell. Cerox1 functions by precisely controlling the levels of at least 12 transcripts coding for subunits of the mammalian mitochondrial complex I, the production of which is suppressed by miR-488-3p [210]. Being the first enzyme of the electron transport chain, complex I mediates the electron transfer from NADH to coenzyme q10, creates a proton gradient across the inner mitochondrial membrane, and produces ROS [211]. The authors of this work demonstrated that the expression of Cerox1, which is conserved across placental mammals, is correlated with an increase in complex I subunit abundance and enzymatic activity (also in the human ortholog CEROX1), a decrease in the production of ROS, and greater protection against the complex I inhibitor rotenone. Given the shared miRNA recognition elements (MRE) in both complex I subunit genes and Cerox1, the effects of miR-488-3p are blocked through the binding of the said miRNA to Cerox1 [210].

All in all, both the significant role of lncRNAs in redox regulation and the contribution of redox in the pathogenesis of disease are becoming more apparent. In fact, in the context of lung adenocarcinoma (LUAD), a novel redox-related lncRNA prognostic signature (redox-LPS) was identified for a more accurate LUAD prognosis. Ren et al. achieved this by including over 700 LUAD samples in their analysis, and a final redox-LPS with four lncRNAs (CRNDE, LINC01137, CASC15 and CYP1B1-AS1) was developed and validated. The authors assessed that the signature they had developed was superior to three other established models in predicting LUAD patient survival [212].

## 5. Oxidative Stress-Induced Formation of Tunneling Nanotubes

### 5.1. Tunneling Nanotubes: Biogenesis and Their Role in Malignancies

Tunneling nanotubes are filamentous actin structures surrounded by a lipid bilayer and serve as intercellular bridges for direct cell-to-cell communication and the transport of various cargoes. They are structurally characterized by their enrichment in F-actin and non-attachment to the extracellular substrate [213]. They have been shown to transfer proteins, RNAs, organelles, and even pathogens between non-adjacent cells [213,214,215,216,217,218,219,220] as well as transduce electrical signals [221]. Organelles demonstrated to be substrates of TNT-based transport include the endoplasmic reticulum, Golgi, endosome, and mitochondria [222].

There are two mechanisms that have been proposed for the biogenesis of TNTs. One posits that cytoplasmic protrusions extend from one cell to a spatially distant cell. The other involves two previously connected cells moving away from one another with the TNTs remaining as intercellular conduits [213,222,223,224].

In cancer, increased communication and interconnectivity are correlated with more aggressive cancer phenotypes. Gliomas, considered the most aggressive brain cancer, have been observed to form a TNT-based network that seems to contribute to increased malignancy and chemoresistance [225]. This correlation has also been observed in other cancer types, such as breast and ovarian cancers.

TNT-mediated communication can be a driver of tumor heterogeneity, which can be observed on different levels. The first concerns inter- or intra-tumoral genetic heterogeneity given the variation in the mutational landscape across either a tumor or different tumors. The second is cellular heterogeneity referring to the mixing/combination of different non-tumor and tumor cells that interact within a cancer. Finally, heterogeneity can also be observed in the tumor microenvironment, which refers to the aggregate of the cells, stroma, blood vessels, and matrix that provides certain niches for specific cell types [226,227]. TNTs can also mediate the horizontal transfer of oncogenes such as mutant KRAS, an oft-mutated gene in cancer that regulates proliferation, survival, and, relevantly, the formation of cellular protrusions. In addition, TNTs allow for the reprogramming of healthy neighboring cells by tumor cells, increasing the likelihood of creating a tumor niche. Their cargo could be used to affect various cancer hallmarks in adjacent cells. Non-coding RNAs such as miRNAs can also be transferred through TNTs and are able to induce more aggressive behavior in the recipient cells [228,229].

Another cancer hallmark affected by TNTs is the tumor microenvironment, which includes stromal cells such as fibroblasts, perivascular cells, endothelial cells and several immune cells such as macrophages, mast cells, and their secreted cytokines and chemokines. The cancer cells’ interaction with the ECM and neighboring cells is key in carcinogenesis, since these also modulate cancer cell survival, angiogenesis, ECM remodeling, invasion, and metastasis. For instance, leukemia cells of an acute lymphoblastoma were shown to communicate with bone marrow stromal cells (BMSCs) via TNTs, inducing the secretion of survival-promoting cytokines [218]. Activated stromal cells were also shown to transfer mitochondria to acute lymphoblastic leukemia cells to rescue them from oxidative stress [230]. 

TNTs also play a role in chemoresistance. In MCF-7 breast cancer cells, cytotoxic doses of 5-fluorouracil significantly induced TNT formation thus aiding in their survival [231]. In pancreatic cancer cells, treatment with doxorubicin stimulated an increased formation of TNTs, which facilitated the intercellular redistribution of the drug between the connected cells. This was supported by visual evidence in vivo obtained via multiphoton fluorescence microscopic imaging of TNTs in tumor specimens resected from three human patients with pancreatic adenocarcinoma, and one with neuroendocrine carcinoma [232].

### 5.2. TNTs and Oxidative Stress

In addition to their roles in cell-to-cell communication and metastasis, TNTs play a role in maintaining homeostasis amidst oxidative stress, and this is supported by the observation that ROS promote TNT formation [233,234,235,236]. Due to the actin-filled nature of TNTs [237], ROS are able to propagate their effects on TNT biogenesis mainly by altering actin dynamics. Among the actin-related parameters that are sensitive to ROS levels/oxidative stress are filament assembly, polymerization, branching, and cytoskeletal reorganization [238,239]. Due to the efficacy of the numerous antioxidant systems discussed above, few studies discuss the direct oxidation of actin by ROS.

Greater focus has been directed at ROS-initiated signaling and how these cascades govern the kinetics of actin protrusion. For instance, H_2_O_2_ was found to stimulate TNT formation via the colocalization of myosin Va and F-actin both within the same cell type and between different cells when co-cultured, likely through the H_2_O_2_-induced phosphorylation of ERK1/2 and P38 MAPK [240]. An interesting observation was made by Abounit et al., who noticed that the transport of alpha-synuclein through TNTs came with an increase in ROS, suggesting that this is due to the higher number of TNTs caused by greater ROS levels [241]. In our recent work on the CSE-upregulated lncRNA SCAL1, we observed that TNTs were more prominent and more readily formed in CSE-treated A549 lung cancer cells, particularly at higher concentrations, hinting at a potential mechanism to adapt to the oxidative stress caused by smoking [57]. Even UV light, serum depletion, and hypoxia have been demonstrated to stimulate TNT formation [235,242,243].

It is likely that TNT formation is heavily regulated by the Rho family of GTPases. Its members, Rac1, Cdc42, and RhoA, have been shown to have a role in TNT biogenesis. Cdc42 and Rac1, as well as their downstream targets, WAVE2 and WASP, respectively, were found to modulate actin polymerization through the Arp2/3 complex, thus affecting TNT biogenesis [244]. To be precise, Cdc42 is involved in the initiation of TNT formation while Rac1 was observed to be found throughout the TNT structures. Moreover, upon the pharmacological inhibition of Rac1 or Cdc42, a decrease in TNT number and lifetime was observed [244].

Aside from controlling actin cytoskeleton and plasma membrane structure formation, these Rho GTPases are also involved in regulating apoptosis. Rac1-regulated oxidase has been found to modulate the production of ROS, inflammation, and programmed cell death in hepatic ischemia [245]. In neurons, which heavily rely on tight control of apoptosis, Rac1 is able to inhibit apoptosis by interfering with downstream signaling of NFκB, PAK, and ERK via ROS-related pathways [244]. The upregulation of the anti-apoptotic BCL2 family of proteins in cancer cells has been shown to be induced by Rac1-mediated MAPK/ERK and Akt signaling [243]. Thus, these Rho GTPase-related signals, through the creation of tunneling nanotubes, are able to suppress apoptosis. Given that most ROS are generated in cells by the mitochondrial respiratory chain [246], it is not surprising that one of the main mechanisms through which TNTs are able to ameliorate oxidative stress and inhibit programmed cell death is through the transfer of functional mitochondria between different cell types.

A greater emphasis, however, has been placed on the role of TNT-mediated mitochondrial transfer under pathological conditions. As shown by Marlein et al., mitochondrial transfer was found to increase ATP production and proliferation rate in vitro and in vivo, at least in the context of BMSCs and multiple myeloma cells [247]. Mitochondrial export from BMSCs to cancer cells also led to improved mitochondrial function, resulting in an enhanced proliferative capacity and invasiveness [247]. 

More relevantly, the shuttling of mitochondria rescues cells from extreme oxidative stress. Human-induced pluripotent stem cell-derived (iPSC) mesenchymal stem cells (MSCs) were demonstrated to alleviate cigarette smoke-induced lung damage and smoke-induced ATP depletion via mitochondrial shuffling through TNTs. Specifically, MSCs were shown to transfer healthy mitochondria to BEAS-2B bronchial epithelial cells to combat cigarette smoke-induced oxidative stress [248].

The acquisition of chemoresistance after mitochondrial transfer in the context of cancer has also been reported, and this is expected given that most chemotherapeutics either elevate ROS levels within cells or alter the cellular redox status [4,249]. Pasquier et al. first showed this in 2013, as they found that mitochondrial transfer from epithelial cells to MCF-7 breast cancer cells granted the latter chemoresistance to doxorubicin, one of the few anthracyclines that are said to generate the highest levels of cellular ROS [250]. Meanwhile, cytarabine, methotrexate, and daunorubicin were rendered less effective due to the shuttling of mitochondria between acute lymphoblastic leukemia (ALL) cells and MSCs in a couple of studies [230,251].

It seems, however, that ROS can also directly promote mitochondrial transfer itself. BMSCs within the protective microenvironment of acute myeloid leukemia (AML) were demonstrated to transfer their mitochondria to AML blasts via AML-derived TNTs [247]. Interestingly, NOX2-derived superoxide coming from the AML increases oxidative stress on the BMSCs, forcing these BMSCs to transport their mitochondria to the AML. When NOX2 was knocked down, superoxide production was lowered, and AML cells were shown to have reduced cellular uptake of mitochondria [247]. Aside from NOX2, a Rho-GTPase known as Miro-1, which has a unique second GTP-binding domain in lieu of the membrane-binding C-terminal domain CAAX motif, has been shown to increase the mitochondrial transfer capacity upon Miro-1 overexpression, resulting in the concomitant reduction in apoptosis and ROS levels [215].

As reviewed by Rustom et al., these findings, when viewed together, depict a strong association between ROS levels, TNT-based supercellularity, and the intercellular shuttling of materials in health and disease. Based on current knowledge about nanotube formation, intercellular transfer and communication phenomena, and the associated molecular pathways, a three-stage framework was proposed, describing ROS-dependent TNT formation that results in redox/metabolic rescue followed by the isolation and removal of degenerate cells [25].

The first step of TNT response to above average levels of ROS is highly dependent on AGE–RAGE signaling. Functioning as “distress signals”, advanced glycation end products (AGEs) are secreted by cells experiencing local stress due to the generated ROS [252]. A prime example of an AGE is S100A4, which is found both extracellularly and intracellularly in both the cytoplasm and nucleus [253,254]. In astrocytes, high concentrations of S100A4 induce TNT outgrowth, but S100A4 has also been found to contribute to enhanced metastasis [252,255]. On the other hand, receptors for advanced glycation end-products (RAGEs) are S100A4 cell surface receptors. As part of the immunoglobulin super family of receptors, RAGEs are known to also participate in inflammatory processes [256].

Upon experiencing oxidative stress, such as through cigarette smoke, S100A4 is reported to be secreted by the stressed cells [257]. The released S100A4 then binds to RAGE, causing an uptick in cytoplasmic ROS production, which then thought to regulate the necessary genes and pathways [258]. This AGE–RAGE interaction is also reported to initiate a self-amplifying loop between sender and receiver that helps identify optimal locations for TNT formation [252]. However, actin-based TNT (AC-TNT) biogenesis, to be distinguished from the formation of microtubule-containing TNTs (MT-TNTs), is believed to only proceed once a defined ROS level is attained [25]. 

In the cells that initiate TNT formation, it is believed that p53 activates caspase-3, which then reduces the S100A4 intracellular concentration via S100A4 cleavage, establishing a gradient (i.e., a higher extracellular concentration of S100A4) that establishes the directionality of TNT formation, thereby helping ensure that TNTs are not directed towards pathological/stressed cells [252]. The conditions of the surrounding stroma, specifically the extracellular matrix, are considered to be a factor in TNT formation. This is based on data showing the induction of filopodia-like protrusions upon the overexpression of hyaluronan synthase 3 [25].

During TNT formation, the mammalian Ste-20-like protein kinase 1 (Mst 1) is thought to be involved; upon stimulation by cROS, it links AGE–RAGE signaling and redox homeostasis with the modulation of cytoskeletal dynamics and, potentially, apoptosis [25]. MST1 is posited to interact with p53 and Akt, with Akt delaying progression into irreversible programmed cell death until necessary [27,259,260]. Several other actin cytoskeleton-related genes mentioned earlier, such as CDC42, ARP2/3, and others, are said to contribute to this stage of the TNT-based response [261]. At this stage, the nascent actin-based TNTs are said to facilitate the distribution of materials among cells to oppose the initial oxidative stress [25]. Passive diffusion along these TNTs allows nutrients, plasma membrane components, RNAs, signaling molecules, ions, and even smaller organelles to cross cellular boundaries. Energy is actively consumed, however, in the myosin motor-mediated bidirectional transport of bigger substrates such as organelles and protein complexes. Notably, mitochondria were not found inside AC-TNTs between cultured PC12 cells, possibly due to the tight wrapping of their actin backbone by the plasma membrane [262]. However, this finding must be scrutinized due to the difficulty of the in vivo examination of these TNTs; these AC-TNTs are believed to be temporary, only lasting for several minutes up to a few hours. As well, the scale of their variability between cell types and developmental stages is still unclear [25].

While upregulated AC-TNT formation can initially counterbalance rising ROS levels, MT-TNTs become more prevalent at states of higher oxidative stress, suggesting that MT-TNTs are involved in a more severe response [25,262]. These MT-TNTs are differentiated from the canonical AC-TNTs through at least three features, aside from an additional detyrosinated microtubule core: a bigger diameter, decreased membrane fluidity due to the oxidation of unsaturated phospholipids, and, lastly, a longer lifetime [262]. 

While the pathways and mechanisms behind MT-TNT formation have not been fully elucidated, it has been hypothesized that AC-TNTs are modified when excessive ROS levels are reached [25]. A pathway potentially governing this modification may involve Mst1, which activates fork head box O3 (FOXO3) and Bcl-2-like pro\tein 11 (Bim), generating mitochondrial ROS (mROS). The accumulation of mROS depolarizes the mitochondrial membrane, triggering the release of cytochrome c, which activates caspase-3, a known effector of apoptosis [263]. However, caspase-3 negatively regulates Bim initially, resulting in the transient inhibition of apoptosis until necessary [264]. The microtubule backbone of MT-TNTs contributes to their greater stability, lifetime, and efficiency in the intercellular transport of material. More importantly, via microtubule-specific motor proteins and/or the intrinsic capacity of microtubules, larger organelles can now be moved, especially mitochondria [25].

At this stage, AGE–RAGE signaling does not seem to play an integral part. This is suggested by the formation of TNTs under oxidative stress in RAGE(−/−) knockout mice and in Chinese hamster ovary (CHO) cells, possibly due to the breaching of the ROS level threshold [236,252]. As well, it was observed in CHO cells that TNT outgrowth is undirected and independent of S100A4, suggesting that these TNTs might have formed due to dysregulation [252]. If neither AC-TNT nor MT-TNT rescue responses can adequately deal with the rising oxidative stress, the intrinsic apoptotic pathway is activated. Here, in this final stage of the framework, the unhealthy cells are isolated and removed from the collective [25]. TNT connections break down so that the healthy cells may be spared from receiving apoptotic signals from the dying cells. In fact, in T lymphocytes, the pro-apoptotic Fas ligand can be transmitted via TNTs to induce death, suggesting a potential mechanism through which unhealthy cells could be disconnected from the network [217,259].

The mechanisms surrounding the uncoupling of TNTs, as well as the determination of how long they will last, is not yet clear, although one main hypothesis has been raised [25]. This involves a ‘passive’ scenario, in which membrane rupture occurs as a consequence of membrane ruffling, the remodeling of cytoskeletal components, or cellular movements. However, the importance of this process rather implies the involvement of precise regulation. In this context, cholesterol plays the essential role of modulating the interrelated properties of the plasma membrane such as lipid organization, phase behavior, membrane fluidity, and mechanical strength [260]. This is highlighted in the finding that increasing concentrations of oxidized cholesterol derivatives, known as oxysterols, affect not only plasma membrane fluidity and strength, but also the activity of membrane receptors and enzymes [265]. Additionally, a higher concentration of cholesterol-rich lipid rafts was observed in TNT-forming mesothelioma [228]. Likewise, depletion of cholesterol has been demonstrated to affect both TNT numbers and apoptosis, the latter via Akt inactivation [266,267].

**Figure 2:**
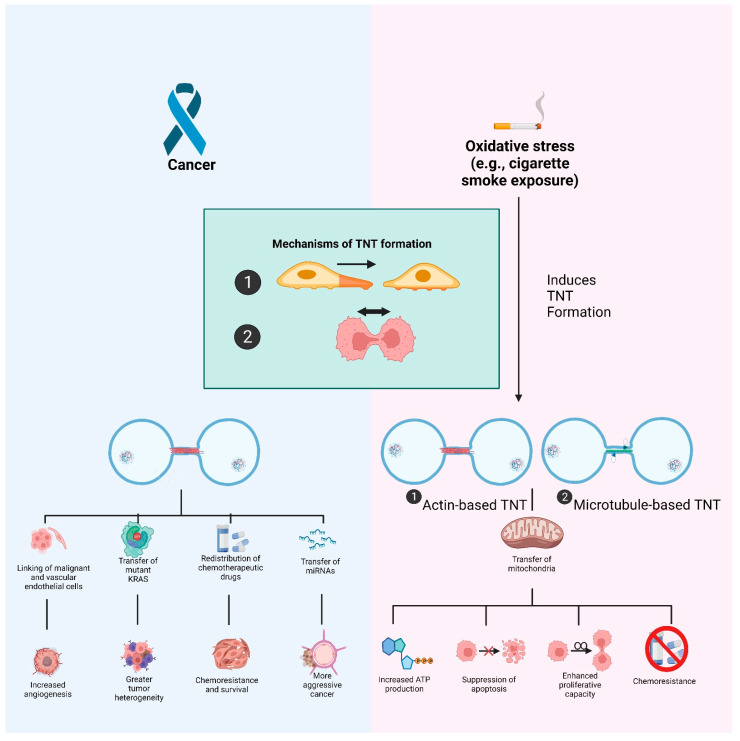
Overview of the role of tunneling nanotubes (TNT) in cancer as well as during oxidative stress. Both contexts involve two proposed mechanisms of TNT formation. In cancer, TNTs are able to transport different cargoes and form connections between cells or cell types to promote and spread cancer phenotypes. During oxidative stress, such as when cells are exposed to cigarette smoke, TNT formation is induced. In particular, the shuttling of mitochondria from healthy cells to cells experiencing a redox imbalance is able to rescue the struggling cells.

The functions of TNTs in cancer and during oxidative stress, as well as their biogenesis, are depicted in Figure 2. 

## 6. Conclusions

ROS are by-products of cell metabolism and play an important role in signaling pathways. Excessive amounts, however, can inflict damage to cells. One exogenous source of ROS that is also tightly linked to cancer is cigarette smoke. As a rich source of known and suspected carcinogens, cigarette smoke can damage both DNA and the epigenome via multiple mechanisms. Although DNA repair systems can restore DNA integrity, incessant toxic insults allow mutations to be propagated. Oxidative stress secondary to cigarette smoke exposure compounds its deleterious effects and may also lead to both mutations and the altered function of DNMTs [41,42,43]. 

An often-underappreciated consequence of the cooperative damage inflicted by cigarette smoke and ROS is the ability of cells to restore ROS homeostasis to evade cell death, even if the damage to oncogenes and tumor suppressor genes may already be beyond repair. In this review, the established role of antioxidant systems in resetting the redox status was revisited. In particular, this review discussed the graded adaptive responses of cells made possible by multi-level transcriptional control [52,53,54] and highlighted the different mechanisms accessed by cells depending on how overwhelmed the antioxidant systems have become.

More importantly, the review included the emerging contribution of lncRNAs and TNTs in regulating oxidative stress. Several lncRNAs are known to regulate the NRF2–KEAP1 pathway itself [58,59,201] while others are known to regulate the inflammatory process [209] or mitochondrial oxidative phosphorylation [210]. With the widely documented roles of lncRNAs in oncogenesis, their regulatory role in antioxidant systems further highlight oxidative stress as an emerging cancer hallmark. 

The involvement of TNTs in regulating redox homeostasis constitutes one of the most significant findings in oxidative stress research. ROS themselves promote TNT formation. TNTs are then able to mediate the intercellular redistribution of ROS species including those induced by chemotherapeutics [232]. The shuttling of mitochondria via TNTs has also been shown to rescue cells from extreme oxidative stress [248]. An apparently graded TNT response also exists. Rising ROS levels are initially counterbalanced by actin-based TNTs, while microtubule-containing TNTs seem to be involved in the more severe oxidative stress response [25,262].

The mechanisms to reset the redox state of cells continue to be unraveled and the repercussions are vast. The adaptive, graded, and transient nature of oxidative stress responses makes redox state manipulation a challenging and difficult therapeutic target.

## Data Availability

Not applicable.
